# MTA2在非小细胞肺癌中的表达及意义

**DOI:** 10.3779/j.issn.1009-3419.2010.08.05

**Published:** 2010-08-20

**Authors:** 术华 王, 艳丽 齐, 俊毅 张, 清富 张, 海英 李, 雪杉 邱

**Affiliations:** 1 110001 沈阳，中国医科大学基础医学院病理学教研室 Department of Pathology, College of Basic Medical Sciences, China Medical University, Shenyang 110001, China; 2 124010 盘锦，盘锦职业技术学院病理学教研室 Department of Pathology, Panjin Vocational and Technical College, Panjin 124010, China; 3 124010 盘锦，盘锦辽河油田第二职工医院病理科 Department of Pathology, the Second Worker's Hospital of Panjin Liaohe Oilfield, Panjin 124010, China; 4 024000 赤峰，赤峰学院医学院病理教研室 Department of Pathology, the School of Medicine, Chifeng College, Chifeng 024000, China

**Keywords:** 肺肿瘤, MTA2, 侵袭, 转移, Lung neoplasms, MTA2, Invasion, Metastasis

## Abstract

**背景与目的:**

已有研究发现转移相关蛋白2（metastasis-associated protein 2, MTA2）在多种肿瘤细胞系中表达且与肿瘤侵袭转移密切相关。本研究旨在研究MTA2在非小细胞肺癌（non-small cell lung cancer, NSCLC）中的表达，并探讨MTA2表达与临床病理特征的关系。

**方法:**

采用免疫组织化学（SP）方法检测110例非小细胞肺癌标本及34例癌旁肺组织中MTA2蛋白表达，并统计分析其表达与NSCLC临床病理特征关系。

**结果:**

MTA2在癌旁肺支气管上皮和肺泡上皮中无表达，在部分NSCLC中呈阳性表达。110例NSCLC标本中MTA2阳性表达率为58.18%（64/110)，MTA2阳性表达与NSCLC的分化程度呈负相关，与临床分期、淋巴结转移呈正相关（*P* < 0.05），与年龄、性别、NSCLC的病理分型无明显相关性（*P* > 0.05）。

**结论:**

MTA2蛋白在部分NSCLC中呈阳性表达且与其分化程度、临床分期、淋巴结转移密切相关，提示肺癌的发生发展可能与MTA2有关，MTA2可能是肺癌新的标志物及治疗靶点。

## Abstract (en)

**Background and objective:**

It has been proven that MTA2 was expressed in many tumor cell lines and was correlated with tumor invasion and metastasis. The aim of this study is to investigate the expression of MTA2 protein in non-small cell lung cancer (NSCLC) and to explore the relationship between the expression of MTA2 and the pathological features.

**Methods:**

The expression of MTA2 protein in 110 cases of NSCLC and 34 cases corresponding lung tissues was detected with immunohistochemistry method. The relationship between the expression of MTA2 and the pathological features in NSCLC was analyzed statistically.

**Results:**

There was no expression of MTA2 protein in cancer collateral branch trachea epithelial and alveolar epithelial, but there was some expression in partial NSCLC. The positive rate of MTA2 was 58.18% (64/110) in 110 NSCLC cases. The expression of MTA2 had negative correlation with differentiation degree of NSCLC but had positive correlation with clinical stage and lymph node metastasis (*P* < 0.05), moreover, it had no obvious correlation with age, gender and pathological type (*P* > 0.05).

**Conclusion:**

There was some expression of MTA2 in partial NSCLC and it was correlated with differentiation degree, clinical stage and lymph node metastasis. The results show that the occurrence and development of lung cancer may be related with MTA2. MTA2 may act as a new symbol and target in the treatment of lung cancer.

转移相关基因2（metastasis-associated gene 2, MTA2）是肿瘤转移相关基因（MTAs）家族成员之一^[1]^，其编码的蛋白MTA2（metastasis-associated protein 2）是具有核小体重塑活性的组蛋白去乙酰基酶（nuclesome remodeling deac-etylase, NuRD）的亚基，其参与组成的复合物具有相对保守的基因转录抑制功能，与生物个体发育有关。MTA2蛋白在多种上皮源性肿瘤细胞系中表达上调，如卵巢癌^[2]^、乳腺癌^[3]^、肝癌等^[4]^，并与肿瘤侵袭转移有关，但其在非小细胞肺癌（non-small cell lung cancer, NSCLC）中的表达情况尚未见报道。本实验用免疫组织化学方法观察MTA2在NSCLC组织中的表达情况，分析MTA2的表达与肺癌临床病理特征的关系，为NSCLC中MTA2的研究积累更多的资料。

## 1 材料与方法

### 1.1 临床资料

110例NSCLC组织来源于2006年1月-2007年12月中国医科大学附属第一临床医院手术切除的标本，全部病例均有完整的临床病理资料。其中男性57例，女性53例; 年龄36岁-78岁，中位年龄57岁; 根据国际抗癌联盟（2002）TNM分期标准分为Ⅰ/Ⅱ期65例，Ⅲ/Ⅳ期45例; 鳞癌48例，腺癌62例; 高中分化46例，低分化64例。34例癌旁肺组织取自手术断端，距癌灶边缘 > 5 cm。所有患者术前均未接受过任何放化疗。

### 1.2 研究方法

采用免疫组织化学（SP）法。羊抗人MTA2多克隆抗体（SC-9446）购自美国Santa Cruz公司; 二抗试剂盒（ZB-2306）、DAB显色试剂盒（ZLI-9032）购自北京中杉金桥生物技术有限公司; SP超敏试剂盒（KIT-9709）购自福州迈新生物技术开发公司。

### 1.3 结果判定

所有切片均由两位有经验的病理研究者在不知道临床参数的情况下独立阅片并计数细胞，结果不统一时，采用两位研究者的综合分析意见。MTA2表达主要定位于癌细胞核中，部分胞质略有表达，呈棕黄色颗粒状。每张切片在200倍显微镜下随机选取10个不同视野，每个视野计数100个细胞。切片内阳性细胞数 > 10%为MTA2阳性表达。

### 1.4 统计学分析

应用SPSS 17.0统计分析软件，对MTA2表达和临床病理特征的关系用*Pearson*相关分析，*P* < 0.05为有统计学差异。

## 2 结果

### 2.1 MTA2的表达情况

MTA2阳性信号主要表达于癌细胞核，阳性表达率为58.18%（64/110）。MTA2在癌旁细支气管上皮和肺泡上皮中无表达，MTA2在高分化鳞癌和腺癌中低表达，在低分化鳞癌和腺癌中高表达（图 1）。

**1:**
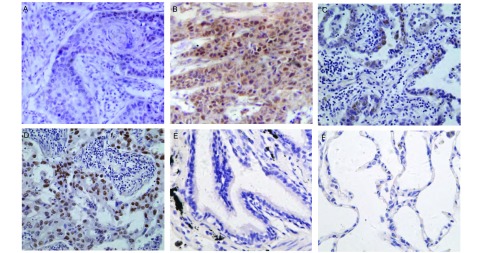
MTA2在NSCLC及其癌旁肺组织中的表达（SP法，×400）。A：高分化鳞癌; B：低分化鳞癌; C：高分化腺癌; D：低分化腺癌; E：癌旁细支气管上皮; F：癌旁肺泡上皮。NSCLC：非小细胞肺癌。 The expression of MTA2 in NSCLC and peficancerous lung tissues (SP, ×400). A: higher-differentiated squamous cell carcinoma; B: lowerdifferentiated squamous cell carcinoma; C: higher-differentiated adenocarcinoma; D: lower-differentiated adenocarcinoma; E: cancer collateral branch trachea epithelial; F: cancer collateral alveolar epithelial. NSCLC: non-small cell lung cancer.

### 2.2 MTA2的表达与NSCLC患者临床病理特征的关系

110例NSCLC组织中有64例MTA2阳性表达，其表达与NSCLC患者临床病理特征之间的关系见表 1。经*Pearson*相关分析，结果显示：MTA2的表达与NSCLC的分化程度呈负相关（*r*=-0.440, *P* < 0.001），与临床分期（*r*=0.256, *P*=0.007）和淋巴结转移（*r*=0.299, *P*=0.001）呈正相关，而与患者的年龄、性别、组织学分型无相关性。

**1:** MTA2表达与NSCLC患者临床病理因素之间的关系 Relationship between expression of MTA2 and clinico-pathological factors in NSCLC

Characteristics	*n*	Expression of MTA2		*r*	*P*
		+	-		
Age				-0.003	0.972
≥55	60	35	25		
< 55	50	29	21		
Gender				-0.031	0.749
Male	57	34	23		
Female	53	30	23		
Histological type				0.034	0.721
Squamous cell carcinoma	48	27	21		
Adenocarcinoma	62	37	25		
Differentiation				-0.440	< 0.001
Highly and moderately differentiated	46	15	31		
Poorly differentiated	64	49	15		
Lymphoid node metastasis				0.299	0.001
N0	50	21	29		
N1-2	60	43	17		
P-TNM stage				0.256	0.007
Ⅰ/Ⅱ	65	31	34		
Ⅲ/Ⅳ	45	33	12		

## 3 讨论

参考文献MTA2是Zhang等^[1]^于1999年发现的肿瘤转移相关基因，定位于染色体11q1213.1，其编码的蛋白质MTA2含668个氨基酸，分子量为70 kDa。与MTA1蛋白有65%的同源性，N端高度相似，但C端的不同之处在于MTA2蛋白缺少myb区和SH3结合区^[5]^。MTA1组成的复合体包含HDAC1/2、RbAp46/48和MBD3等组件，而MTA2组成的复合体则包括Sin3和Mi2组件，此外，MTA2组成的复合体与HDAC1复合体较相似，提示MTA2可能具有内部处理作用^[6, 7]^。MTA2蛋白也是核小体重塑活性的NuRD的亚基^[7]^。目前认为MTA2可通过调节雌激素通路^[8-10]^、细胞骨架^[11]^和细胞凋亡^[12]^等途径促进肿瘤的侵袭和转移。

已有研究^[2, 3, 10]^证实，MTA2在恶性肿瘤和其来源的正常组织中的表达存在差异，在同种肿瘤中有淋巴结转移的和无淋巴结转移的表达存在差异。Ji等^[2]^利用免疫组织化学技术检测了不同分组的卵巢癌标本中MTA2的表达，结果发现临床分期Ⅲ/Ⅳ期的组织表达水平高于Ⅰ/Ⅱ期; 有淋巴结转移组表达水平高于无淋巴结转移组; 低分化组表达水平高于高分化组。RT-PCR和Western blot技术检测也有相同趋势，证明MTA2的表达水平与临床分期、淋巴结转移及病理学分级相关。Cui等^[3]^发现MTA2还可促进雌激素受体依赖的乳腺癌细胞的生长。最新研究^[13]^表明MTA2与EGFR、IRAK-1、IkappaBalpha、NFkappaB等因子之间存在交叉对话，其作为转录因子，可参与调控胰腺癌细胞的转移。此外，Luo等^[12]^采用p53和MTA2共转染技术揭示：MTA2以剂量依赖性方式抑制了p53介导的*p21*基因转录激活作用，MTA2影响了p53诱导的细胞生长停止和凋亡的发生。用组蛋白去乙酰基酶复合物（histone deacetylases, HDAC）抑制剂毛癣素A（trichostatin A, TSA）处理可逆转MTA2引起的细胞凋亡的下降，证明MTA2与细胞凋亡密切相关。

Fujitat等^[14]^在研究乳腺癌过程中发现了MTA1、雌激素受体（estrogen receptor, ER）、MTA3、Snail、E-钙粘素通路，在转移性乳腺癌中高表达的MTA1蛋白和HDAC一起，通过与存在于MTA3启动子近端的雌激素反应元件半位点相作用，影响了ER对MTA3的转录激活，下调的MTA3又使得其对Snail的转录抑制作用下降，增多的Snail蛋白最终引起E-钙粘素的表达减少，癌细胞间的粘连减弱易于脱落形成转移。我们对110例NSCLC中MTA2的表达情况及其与NSCLC临床病理特征的关系进行研究，发现MTA2在部分NSCLC中呈阳性表达，表达率为58.18%（64/110）。统计学分析证实MTA2的表达与患者的年龄、性别、NSCLC的组织分型无明显关系，而与肺癌淋巴结转移（*r*=0.299, *P*=0.001）、临床分期（*r*=0.256, *P*=0. 0 0 7）正相关，与分化程度负相关（*r*=- 0. 4 4 0, *P* < 0.001）。实验结果与我们课题组前期实验所证实的MTA1在NSCLC中的表达相类似。因此，推测MTA2可能通过与MTA1相同的作用途径促进肿瘤的转移。

综上所述，MTA2与NSCLC侵袭转移的生物学特性密切相关。进一步深入研究MTA2的作用底物，阻断其作用途径以及发展MTA2的特异性抑制剂来控制肿瘤的发展，将有可能成为临床治疗NSCLC的新靶点。
